# Supplementary material for: Novel Driver Strength Index highlights important cancer genes in TCGA PanCanAtlas patients
Source: PeerJ. 2022 Aug 11;10:e13860. doi: 10.7717/peerj.13860 (PMC9375969; doi:10.7717/peerj.13860)
Supplement: Supplemental Information 2 [file peerj-10-13860-s002.zip › COHORTS/genes plots/cohorts/2021_8_16_14_9_distribution_cohorts_UCEC.pdf]

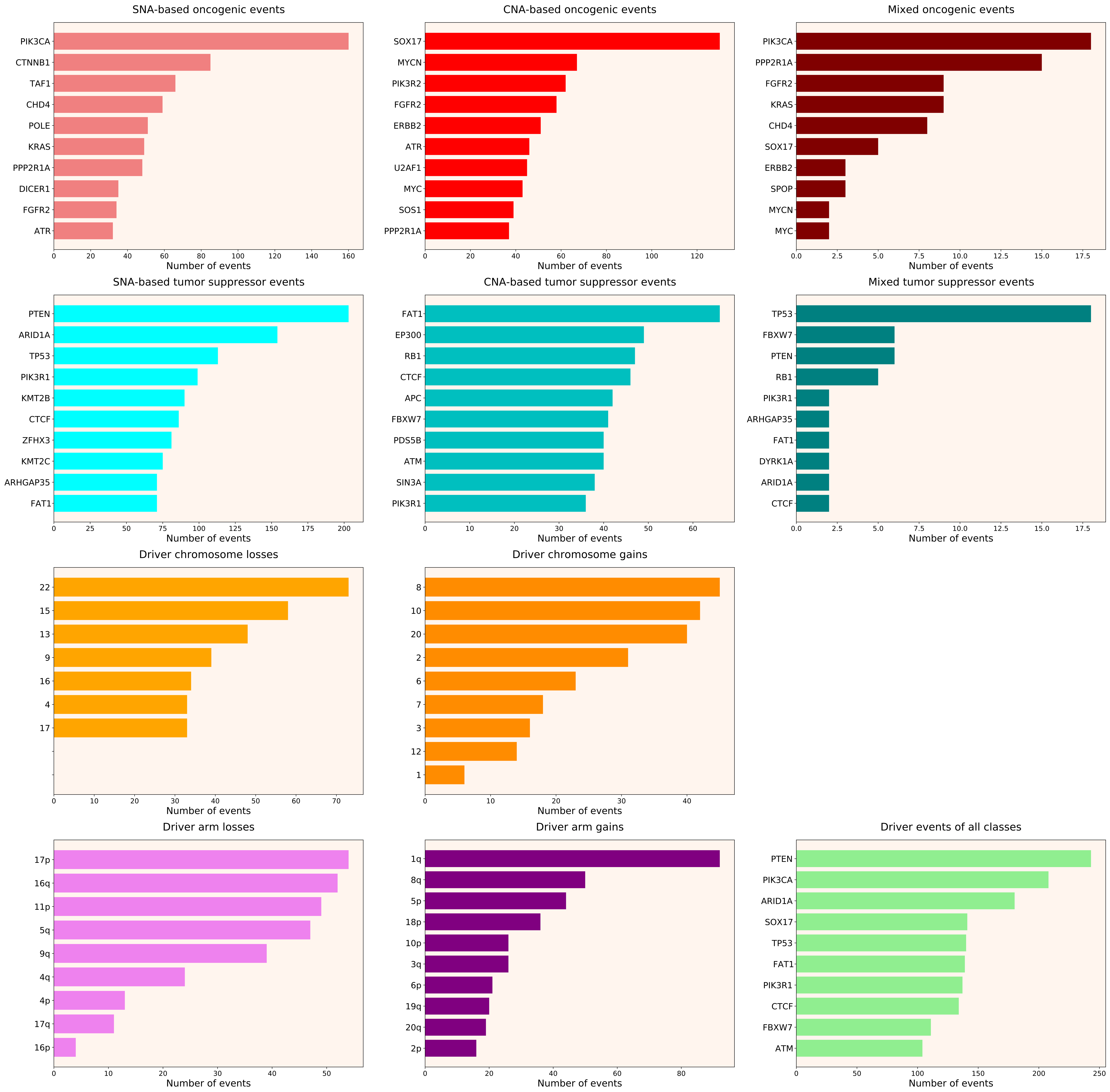
SNA-based oncogenic events
PIK3CA
CTNNB1
TAF1
CHD4
POLE
KRAS
PPP2R1A
DICER1
FGFR2
ATR
0 20 40 60 80 100 120 140 160
Number of events
CNA-based oncogenic events
SOX17
MYCN
PIK3R2
FGFR2
ERBB2
ATR
U2AF1
MYC
SOS1
PPP2R1A
0 20 40 60 80 100 120
Number of events
Mixed oncogenic events
PIK3CA
PPP2R1A
FGFR2
KRAS
CHD4
SOX17
ERBB2
SPOP
MYCN
MYC
0.0 2.5 5.0 7.5 10.0 12.5 15.0 17.5
Number of events
SNA-based tumor suppressor events
PTEN
ARID1A
TP53
PIK3R1
KMT2B
CTCF
ZFHX3
KMT2C
ARHGAP35
FAT1
0 25 50 75 100 125 150 175 200
Number of events
CNA-based tumor suppressor events
FAT1
EP300
RB1
CTCF
APC
FBXW7
PDS5B
ATM
SIN3A
PIK3R1
0 10 20 30 40 50 60
Number of events
Mixed tumor suppressor events
TP53
FBXW7
PTEN
RB1
PIK3R1
ARHGAP35
FAT1
DYRK1A
ARID1A
CTCF
0.0 2.5 5.0 7.5 10.0 12.5 15.0 17.5
Number of events
Driver chromosome losses
22
15
13
9
16
4
17
0 10 20 30 40 50 60 70
Number of events
Driver chromosome gains
8
10
20
2
6
7
3
12
1
0 10 20 30 40
Number of events
Driver arm losses
17p
16q
11p
5q
9q
4q
4p
17q
16p
0 10 20 30 40 50
Number of events
Driver arm gains
1q
8q
5p
18p
10p
3q
6p
19q
20q
2p
0 20 40 60 80
Number of events
Driver events of all classes
PTEN
PIK3CA
ARID1A
SOX17
TP53
FAT1
PIK3R1
CTCF
FBXW7
ATM
0 50 100 150 200 250
Number of events
